# Genome Sequence Analysis of the Fungal Pathogen *Fusarium graminearum* Using Oxford Nanopore Technology

**DOI:** 10.3390/jof7090699

**Published:** 2021-08-27

**Authors:** Zhigang Hao, Yuanyuan Li, Yunyun Jiang, Jiaqing Xu, Jianqiang Li, Laixin Luo

**Affiliations:** Beijing Key Laboratory of Seed Disease Testing and Control, Department of Plant Pathology, College of Plant Protection, China Agricultural University, Beijing 100193, China; haozhigang@cau.edu.cn (Z.H.); liyuanyuan2018@cau.edu.cn (Y.L.); sy20203192999@cau.edu.cn (Y.J.); B20203190880@cau.edu.cn (J.X.); lijq231@cau.edu.cn (J.L.)

**Keywords:** de novo assembly, *Fusarium*, oxford nanopore technology, effector

## Abstract

*Fusarium graminearum* is a plant pathogen of global importance which causes not only significant yield loss but also crop spoilage due to mycotoxins that render grain unsafe for human or livestock consumption. Although the full genome of several *F. graminearum* isolates from different parts of the world have been sequenced, there are no similar studies of isolates originating from China. The current study sought to address this by sequencing the *F. graminearum* isolate FG-12, which was isolated from the roots of maize seedlings exhibiting typical symptoms of blight growing in the Gansu province, China, using Oxford Nanopore Technology (ONT). The FG-12 isolate was found to have a 35.9 Mb genome comprised of five scaffolds corresponding to the four chromosomes and mitochondrial DNA of the *F. graminearum* type strain, PH-1. The genome was found to contain an approximately 2.23% repetitive sequence and encode 12,470 predicted genes. Additional bioinformatic analysis identified 437 genes that were predicted to be secreted effectors, one of which was confirmed to trigger a hypersensitive responses (HR) in the leaves of *Nicotiana benthamiana* during transient expression experiments utilizing agro-infiltration. The *F. graminearum* FG-12 genome sequence and annotation data produced in the current study provide an extremely useful resource for both intra- and inter-species comparative analyses as well as for gene functional studies, and could greatly advance our understanding of this important plant pathogen.

## 1. Introduction

*Fusarium graminearum* is a plant pathogen of global significance which not only causes dramatic yield loss but also reduces grain quality via the production of mycotoxins harmful to both humans and animals [1]. This ascomycete fungus infects many cereal crops, including rice and oats, and is responsible for *Fusarium* head blight (FHB) in wheat and barley as well as *Fusarium* ear blight (FEB) and stem rot in maize. These diseases pose a serious threat to global food production [2]. Indeed, *F. graminearum* is frequently cited as one of the 10 most damaging fungi in agriculture [3]. Unfortunately, the complexity of the host–pathogen interaction and plant resistance mechanisms makes the diseases caused by *F. graminearum* very difficult to control, as there are very few resistant cultivars [4]. It is therefore critical to gain a better understanding of the *F. graminearum*–host interaction in order to develop more effective methods of control.

The characterization of complete genomes is often considered the cornerstone to understanding the biochemical processes underlying host–pathogen interactions, with numerous advantages over partially sequenced genomes. First, complete genomes provide a general understanding of the size, heterozygosity, repetitive sequence content, and other genetic characteristics of a species [5]. Second, complete genomes provide an abundance of data that allow interspecific and intraspecific comparisons which are critical for evolutionary analysis [6]. Third, complete genomes provide a valuable open source database that facilitates all aspects of biological research [7].

The first *Fusarium* genome to be sequenced was that of the *F. graminearum* type strain PH-1, which was originally sequenced using Sanger technology [8]. In subsequent years several other *Fusarium* species, as well as 110 *F. graminearum* strains collected from Europe, Canada, the USA, Brazil, and Australia, were sequenced by various techniques (Tables S1 and S11). However, to date no Chinese isolates of *F. graminearum* have been fully sequenced, even though maize seedling blight (MSB) has become more prevalent in recent years, and the damage caused by FEB and FHB results in huge yield losses in the major maize- and wheat-growing regions of China every year [9,10]. The importance of such regional data cannot be undervalued, as population analyses have shown that the genome of *F. graminearum* can vary greatly among strains [11]. It was reported that 60 diverse *F. graminearum* isolates in North America can be distinguished by dozens of genes with signatures of selection and an array of accessary genes, suggesting that the populations may be equipped with different traits to adapt the agroecosystem [12].

The development of novel single-molecule sequencers, which have the advantage of producing long reads, has brought sequencing technology into a new era [13]. For example, the MinION mini-sequencer from Oxford Nanopore Technology (ONT) has greatly improved the affordability of genome sequencing projects, whilst at the same time providing a more powerful platform for analysis and genome assembly compared to other single-molecule real-time sequencing platforms such as the Pacific Biosciences sequencers [14]. ONT has greatly improved the quality of genome assembly, with the release of full contig maps because of its long reads. This technology is widely used in the sequencing of many species, including *Homo sapiens* [15,16], *Chrysanthemum nankingense* [17], *Phytophthora cinnamomi* [18], *Pseudomonas aeruginosa* [19], and coronavirus [20]. However, although this technology can provide more complete and accurate genomic data, to date none of the *F. graminearum* genomes listed in the NCBI database have been sequenced using this method (Table S1). The genome analysis of 110 *F. graminearum* revealed that most of the genomes were at the contig level, and only 4 were at the chromosome level with some unmapped contigs in NCBI database (Table S1). Given the importance of complete genomes as a resource for further study [21] and the increasing threat of diseases caused by *F. graminearum* in China [22], the generation of a high-quality genome for a Chinese isolate is long overdue. The current study sought to address this issue by using ONT to perform de novo sequencing and genome assembly for a *F. graminearum* isolate, FG-12, which was originally collected from diseased roots of maize seedlings grown in the Gansu province of China. Having achieved this, the study went on to use bioinformatic analysis to identify candidate genes associated with pathogenicity and *Agrobacterium*-mediated transient expression to evaluate putative effector proteins.

## 2. Results

### 2.1. Chromosome-Scale Genome Sequence Assembly

The genome of *F. graminearum* FG-12 was assembled using the genomic DNA sequences produced using ONT, which constituted 8.0 G of data. A total of 536,756 single-molecule nanopore long reads with an average length of 14.9 kb were obtained after data filtering using the Oxford Nanopore Metrichor basecaller. During genome assembly the nanopore long reads were verified using re-sequencing data to correct for errors. The resulting genome was 35.95 Mb in size, which was 89.6% of the estimated genome size (~40.14 Mb) determined by k-mer analysis (Figure S1, Table S2). The N50 contig length was approximately 7.8 MB, and the genome consisted of 5 scaffolds with an overall GC content of 47.99% (Table 1). Compared with other genomes of *F. graminearum* listed in the National Center for Biotechnology Information (NCBI) database, it is a high-quality genome without unmapped contigs.

### 2.2. Genome Annotation

The FG-12 genome data were initially analyzed using MAKER and AUGUSTUS gene prediction programs, which identified many putative coding sequences (CDS). Further bioinformatic analysis utilizing RNA-seq data and homologous sequences from other *Fusarium* species resulted in an ab initio prediction of 12,470 high confidence protein-coding genes encoded by the FG-12 genome. The accuracy of this prediction was further evaluated by Benchmarking Universal Single-Copy Orthologs (BUSCO) analysis using eukaryote, fungi, ascomycota, and sordariomycete library data, which matched 90% of the single-copy FG-12 genes to the four libraries, an indication that the FG-12 genome data was of extremely high quality (Table 2, Figure S2). In addition, Rfam analysis of the FG-12 genome identified 354 non-coding RNA (ncRNA) sequences (Table 2 and Table S4). The mitogenome of FG-12 was 98,312 bp in length, containing 28 protein-coding genes (PCGs), 28 transfer RNA genes (tRNAs), and 4 ribosomal RNA genes (Table 3).

Functional annotation using EggNOG (Table S3) revealed that of the 12,470 predicted genes, 8992 genes (72.11%) could be annotated by EggNOG, and 3565 genes (28.59%) could be assigned GO terms (Figure S3), while 2662 genes (21.35%) could be ascribed to KEGG pathways (Figure S4).

### 2.3. Repetitive Sequence Content

Bioinformatic analysis using RepeatModeler and RepeatMasker software indicated that approximately 2.23% of the FG-12 genome consisted of repetitive elements (Table 4), which is greater than the 1.68% recorded for the PH-1 genome. This discrepancy was observed in nearly all the individual classes of repetitive DNA, although it is interesting to note that the FG-12 genome completely lacked the DNA transposons that made up 0.23% of the PH-1 genome (Table 4). Similar comparison to the *Magnaporthe oryzae* genome revealed that FG-12 had a much lower overall repetitive DNA content (2.23% compared to 11.75%), and only contained the Gypsy/DIRS1 class of long terminal repeat retrotransposons (LTRs), while the *M. oryzae* genome also contained Ty1/Copia LTRs (Table S5).

### 2.4. Gene Synteny Analysis of the F. Graminearum FG-12 and PH-1 Genomes

**Figure 1:**
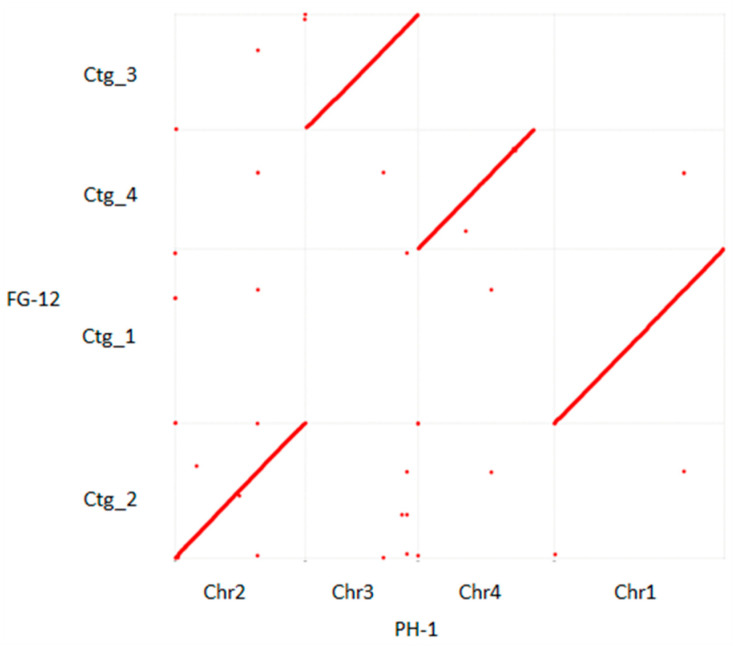
Dot plot diagram comparing the structure of the *F. graminearum* FG-12 and PH-1 genomes. Complete genome alignment using MUMmer software was used to compare the 4 scaffolds of the FG-12 genome (Ctg_1–4) listed on the y-axis to the 4 chromosomes (Chr1–4) of the PH-1 genome on the x-axis. Forward matches are indicated by red dots, while reverse matches are in blue. If the 2 sequences were to be perfectly identical, a single red line would dissect the graph from the bottom left to the top right.

Alignment of the 5 scaffolds comprising the FG-12 genome with the 4 chromosomes and mitochondrial DNA of PH-1 indicated a high degree of homology between the 2 genomes in which the 5 FG-12 scaffolds (Ctg_1–5) were found to correspond to chromosomes 1, 2, 3, 4 and the mitochondrial DNA of PH-1, respectively (Figure 1 and Figure S11). The phylogenetic tree showed that FG12 and PH-1 were on the same clade when the genomes were analyzed with 9 other sequenced filamentous fungi, including 3 other species strains in *Fusarium* and 6 other genera strains (Table S12), using MEGA-X (https://www.megasoftware.net/ accessed on 18 August 2021) (Figure S12). However, the 2 genomes were not identical, with the most notable difference being that scaffold 4 (Ctg_4) of FG-12 was found to lack a small region (about 1.5 Mb in length) located at the carboxyl terminus of chromosome 4 in PH-1. Bioinformatic analysis of the PH-1 chromosome revealed that this region contained a total of 83 genes (Table S6). However, only 73 of these genes could be annotated by EggNOG analysis (Table S7), which indicated that majority were associated with enzyme activator activity (Figure S5), while KEGG annotation revealed that most of these could be ascribed to the thermogenesis pathway associated with environmental adaptation (Figure S6).

### 2.5. Carbohydrate Active Enzymes and Secondary Metabolite Gene Clusters

Analysis of the FG-12 CDS data using the CAZy annotation pipeline identified 501 putative CAZyme genes (Table 5), including 238 glycoside hydrolases (GH), 100 glycosyltransferases (GT), 94 auxiliary activities (AA), 41 carbohydrate esterases (CE), 22 polysaccharide lyases (PL), and 6 carbohydrate-binding modules (CBM). This CAZyme profile was very similar to that of the *F. graminearum* type strain PH-1, which had 522 putative CAZyme genes (Table 5 and Table S6).

**Figure 2:**
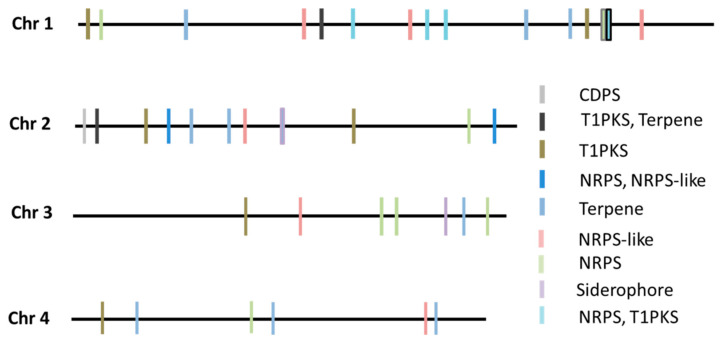
Type and location of secondary metabolite gene clusters (SMC) within the FG-12 genome. NRPS indicates non-ribosomal peptide synthases, while T1PKS indicates type 1 polyketide synthases.

Subsequent analysis using the antiSMASH online tool revealed a total of 39 secondary metabolites gene clusters (SMC) within the FG-12 genome, fewer than the 44 found in PH-1. The FG-12 SMCs were allocated to 9 categories including 1 CDPS cluster, 2 terpene-T1PKS clusters, 7 T1PKS clusters, 1 NRPS-NRPS-like cluster, 9 terpene clusters, 7 NRPS clusters, 6 NRPS-like clusters, 5 T1PKS-NRPS clusters, and 1 siderophore cluster (Figure 2). Further analysis indicated that these putative SMCs contained whole or partial sequences of known biosynthetic gene clusters, including the trichodiene-11-one cluster of *Fusarium asiaticum* (BGC0001811), the gibepyrone-A cluster of *Fusarium fujikuroi* (BGC0001606), the BII-rafflesfungin cluster associated with *Phoma* sp. (BGC0001966), the naphthopyrone cluster from *Aspergillus nidulans* (BGC0000107), the zearalenone cluster of *F. graminearum* (BGC0001057), the fusarielin H cluster from *F. graminearum* (BGC0001600), the oxyjavanicin cluster from *F. fujikuroi* (BGC0001242), the squalestatin S1 cluster associated with *Aspergillus* sp. (BGC0001839), the NG-391 cluster from *Metarhizium anisopliae* (BGC0001026), the koraiol cluster from *Fusarium fujikuroi* (BGC0001642), and the chrysogine cluster from *F. graminearum* (BGC0001545), strongly indicating that FG-12 is capable of synthesizing all of these metabolites.

**Figure 3:**
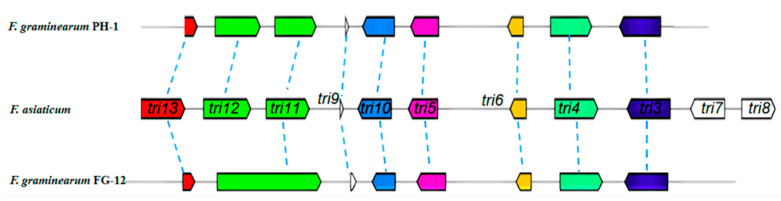
Comparison of the TRI gene clusters of *F. graminearum* FG-12 and PH-1. The trichothecene biosynthetic (TRI) gene cluster of *F. graminearum* FG-12 was found to contain 8 sequences homologous to the TRI genes of *F. graminearum* PH-1 and *F. asiaticum* (BGC0001811). Arrows indicate gene direction.

The current study also made a direct comparison between the trichodiene-11-one cluster of FG-12 and PH-1, a gene cluster known to be responsible for the synthesis of deoxynivalenol (DON), a potent B-type trichothecene mycotoxin that lowers grain quality and poses a serious threat to the heath of both humans and livestock. Sequence alignment using the *F. asiaticum* cluster (BGC0001811) as a bridge sequence revealed that the trichodiene-11-one cluster, also known as the trichothecene biosynthetic (TRI) gene cluster, of FG-12 only contained 8 genes (*tri13* FGMG_005176, *tri11* FGMG_005177, *tri9* FGMG_005178, *tri10* FGMG_005179, *tri5* FGMG_005180, *tri6* FGMG_005181, *tri4* FGMG_005182, *tri3* FGMG_005183), which is 1 less than the 9 genes found in PH-1, and 3 less than *F. asiaticum* (Figure 3). Moreover, the *tri11* gene of FG-12 was found to be much longer than that of the other 2 species, which could indicate that this gene has a different function in FG-12.

### 2.6. Prediction of Putative Effectors

Bioinformatic analysis using SignalP, TMHMM, PredGPI, and Perl identified a total of 437 putative effectors—pathogenicity-related (PR) proteins that are excreted into the extracellular space—by selecting small (≤500 amino acids), cysteine-rich (≥4 cysteine residues) candidate proteins that contained a signal peptide but lacked a transmembrane region or GPI anchor (Table S8) The numbers of total proteins, proteins with signals, proteins without transmembrane domains, and GPI anchors predicted in strain FG-12 were similar to those in strain PH-1, but were slightly lower (Table S13). Subsequent GO annotation of the putative effectors indicated that many were associated with hydrolase activity (Figure S7), while KEGG analysis ascribed the majority to pathways associated with betalain, tryptophan, isoquinoline alkaloid, and melanin biosynthesis (Figure S8).

### 2.7. Functional Analysis of Putative Effectors

**Figure 4:**
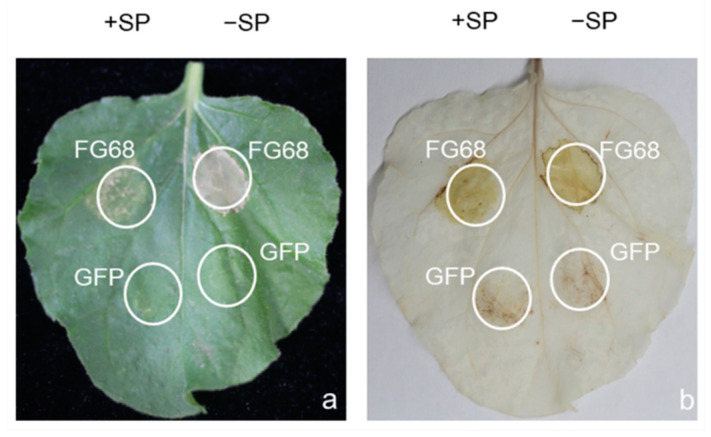
Transient expression of the putative effector FG68 in *N. benthamiana* leaves via agro-infiltration. Leaves of *N. benthamiana* inoculated with *Agrobacteria* carrying FG68 were assessed at 3 days post-inoculation (dpi) for both symptoms of cell damage (**a**), and the presence of hydrogen peroxide via DAB staining (**b**). Expression of green fluorescent protein (GFP) was used as a negative control. +SP and −SP refer to the putative effector with or without signal peptides, respectively.

Secreted effectors are known to play a key role in the infection process of plant pathogenic fungi. The transient expression of five putative FG-12 effectors, respectively based on the CFEM domain (common in several fungal extracellular membrane proteins) [23], as well as ribonuclease domain [24], which are both related to fungal pathogenicity assessed in *N. benthamiana* by agro-infiltration, indicated that only one, FG68 (FGMG_012098), produced visible symptoms of cell damage (Figure 4 and Figure S9). Subsequent DAB staining revealed the presence of high concentrations of H_2_O_2_ associated with the expression of FG68, which encodes a fungal ribosomal nuclease, indicating that this protein could initiate a powerful hypersensitive reaction (HR) in the host (Figure 4).

## 3. Discussion

The development of third-generation sequencing technology over the past 10 years has dramatically improved the capacity to efficiently sequence whole genomes [25]. The ONT sequencing platform in particular provides an affordable method to produce high-quality genome assemblies [26,27,28], and has been applied widely in both prokaryotes [29] and eukaryotes including fungi [30]. However, until now no *F. graminearum* isolates have been sequenced using this technology. The current study involved the reconstruction of the full genome of a Chinese isolate of *F. graminearum*, FG-12, using nanopore long reads. The resulting genome comprised 35,949,582 bp and consisted of 5 scaffolds corresponding to 4 chromosomes and a mitochondrial DNA, which made up the genome of the *F. graminearum* strain FG-12without any unmapped data. Bioinformatic analysis of the FG-12 genome predicted a total of 12,470 high confidence protein-coding gene sequences, of which 8992 could be annotated by EggNOG analysis, 3565 were assigned a GO term, and 2662 were ascribed to a KEGG pathway. The genomic and annotation data produced in the current study therefore represent an extremely useful resource on which to base future research projects.

Although the FG-12 genome exhibited a high degree of homology with the *F. graminearum* type strain PH-1, there were some key differences. For example, the Chinese FG-12 isolate was found to lack a small region (about 1.5 Mb in length) located at the carboxyl terminus of chromosome 4 in the PH-1 genome. This region was found to encode 83 putative genes, many of which were ascribed to KEGG pathways associated with thermogenesis, which is a pathway known to be associated with environmental adaptation [31,32]. In addition, the FG-12 genome had a higher proportion of repetitive DNA than PH-1 (2.23% compared to 1.68%), but lacked the DNA transposons found in the type strain. Such differences provide further evidence of the genetic diversity found within *F. graminearum*, and reaffirm the value of sequencing complete genomes from isolates collected from different regions of the world.

Cell wall-degrading enzymes play a key role in the infection process of plant pathogenic fungi, allowing them to penetrate host tissue while providing a source of nutrition by the release of simple carbohydrates [33]. The current study found a total of 501 putative CAZyme genes within the predicted CDS data from the FG-12 genome, a number slightly lower than the 522 found in the PH-1 genome, and resulted in FG-12 also having a lower number of genes allocated to specific CAZyme categories including GH, AA, CE, and CBM. Interestingly, this was not in the case for PL and GT. Many of the FG-12 CAZyme genes were found to be homologous with genes known to be virulence factors in other plant pathogens. For example, FGMG_010998-RA (GH) corresponded to PsXEG1 of *Phytophthora parasitica*, which acts as a pathogen-associated molecular pattern (PAMP) that can trigger host defense responses, including cell death [34]. The different CAZyme profiles of FG-12 and PH-1 were also of great interest as they might provide clues regarding the host specificity and disease manifestation associated with different isolates of *F. graminearum*, since FG-12 is most commonly associated with root blight of maize, while PH-1 is associated with FHB in wheat and barley. However, further investigation is required to discover how such CAZyme profiles might affect other *F. graminearum* isolates, as well as to identify the key enzymes responsible.

Secondary metabolites, especially fungal toxins, are also known to play a key role in the pathogenicity of some plant pathogenic fungi including deoxynivalenol (DON), which is a key virulence factor in *F. graminearum* [35]. The current study identified a total of 39 secondary metabolites clusters within the FG-12 genome, which was less than the 44 found in PH-1. As expected, the FG-12 genome was found to contain the trichodiene-11-one biosynthetic gene cluster associated with DON production, but interestingly it was comprised of only 8 genes rather than the 9 genes found in PH-1 and the 11 of *F. asiaticum*, which could either indicate a degree of gene redundancy in the trichodiene-11-one gene cluster, or that the genes have different functions in the three fungi.

Secreted effectors are another class of virulence factor that can influence the host–pathogen interaction [36]. The current study identified 437 putative effectors in FG-12, 5 of which were investigated by transient expression in *N. benthamiana*. However, only one, *FG68*, was found to trigger a significant hypersensitive response. Bioinformatic analysis revealed that *FG68* encoded a secreted RNase similar to Zt6, which is a known virulence factor in *Zymoseptoria tritici* [24], and it was extremely interesting to note that the homologous sequence from PH-1, which lacked a signal peptide, failed to trigger cell death in a previous study [37]. Sequence alignment revealed that the FG-12 and PH-1 sequences differed by only four amino acid residues (Figure S10). Taken together, these findings provide further evidence of how genetic variation can affect the pathology of *F. graminearum*, and again highlight the utility of having complete genome sequences from different isolates.

In conclusion, in the current study a high-quality complete genome sequence for *F. graminearum* FG-12 was successfully produced using ONT, constituting the first complete genome for a Chinese isolate. Genome annotation and preliminary gene verification experiments went on to identify key differences between FG-12 and the *F. graminearum* type strain PH-1, demonstrating that the genomic sequences and annotation data produced in the current study provide an extremely useful resource for the future study of this important plant pathogen.

## 4. Materials and Methods

### 4.1. Strains and Culture Conditions

The *F. graminearum* isolate used in the current study, FG-12, which was originally collected from diseased roots of maize seedlings growing in the Gansu province of China, was selected because it produces very aggressive infections in maize roots and is the most commonly used laboratory strain in China. The FG-12 isolate was routinely maintained on potato dextrose agar (PDA) at 25 °C. The strain (available upon request) is deposited at the Seed Health Centre of China Agricultural University, Beijing, China. Routine cloning procedures were performed using *Escherichia coli* DH5α cultured on Luria–Bertani (LB) medium at 37 °C, while the *Agrobacterium tumefaciens* strain, GV3101, which was used in the functional analysis experiments, was grown on LB medium at 28 °C. The *Nicotiana benthamiana* plants used in the functional analysis were grown in pots kept in a greenhouse, which was maintained at 25 °C with a 16 h photoperiod and watering when necessary.

### 4.2. Total DNA and RNA Extraction

Genomic DNA was extracted using a Blood and Cell Culture DNA Midi Kit (Cat. No.13343, Qiagen, New York, NY, USA) in accordance with the instructions of the manufacturer. The concentration and purity of the resulting DNA were determined using a Qubit fluorometer and nanodrop 2000 spectrophotometer (Thermo Fisher Scientific, Carlsbad, CA, USA), while the DNA integrity was assessed by electrophoresis using a 0.5% agarose gel.

Fungal RNA was extracted using the TRIzol reagent (Invitrogen, Carlsbad, CA, USA) following the protocol of the manufacturer. The concentration of the resulting RNA samples was determined using the Nanodrop system (NanoDrop, Madison, WI, USA), and its integrity verified by its RNA integrity number (RIN), which was obtained using an Agilent 2100 Bioanalyzer (Agilent, Santa Clara, CA, USA).

### 4.3. Library Construction and Sequencing

The genomic sequencing was performed using the ONT PromethION P24 system at the Beijing Genomics Institute (BGI) located in Shenzhen, China. Prior to sequencing, a genomic DNA library was constructed using the Ligation Sequencing Kit (SQK-LSK109) and a Native Barcoding Kit (EXP-NBD114) in accordance with the standard 1D Native Barcoding protocol provided by the manufacturer (Oxford Nanopore Technology, Oxford, UK). The resulting library was quantified using the Qubit DNA HS Assay Kit and a Qubit fluorometer (Thermo Fisher Scientific, Bedford, MA, USA) before being loaded into the Flow Cell R9.4.1 of the PromethION P24 device (BGI, Shenzhen, China) to obtain the ~220× data via the ONT platform.

In addition to the nanopore sequencing, ~40× short reads with insert sizes of 300–400 bp were generated using the BGISEQ platform (BGI, Shenzhen, China) for use in genome polishing, while the 47 M RNA-seq reads used in the gene prediction analysis were produced using the BGIseq500 platform (BGI, Shenzhen, China).

### 4.4. Genome Assembly

Before genome assembly, the size of the *F. graminearum* FG-12 genome was estimated by performing k-mer (k = 19) analysis using Jellyfish software [38], which resulted in an estimated genome size of ~40 M. The filtered nanopore long reads were then used as the input for genome assembly using wtdbg2 (v2.5) [39] software in wtpoa-cns mode with the following parameters: -t 6 -x ont -g 37 M -L 500 -l 500 -e 2 –i FG-12. filtered_reads.fq.gz –o FG-12. After completing the initial assembly, iterative polishing was performed using Pilon (v1.23) software in which the filtered paired-end reads were aligned with the raw assembly [40]. The Pilon program was run with default parameters to fix incorrect bases, fill gaps, and correct for local misassemblies. The mitochondrial genome was assembled into a circular form using GetOrganelle (v1.7.5) software [41].

### 4.5. Structural and Functional Annotation of Genes Encoded by the FG-12 Genome

Putative protein-coding sequences (CDS) in the *F. graminearum* FG-12 genome were predicted using the MAKER software package (v2.31.10) with reference proteins from published *Fusarium* genomes and the transcriptome data generated in the current study [42]. Additional analysis using AUGUSTUS software (v3.03) with the species option set to *F. graminearum* was used to identify predicted gene structures [43]. The predicted gene structures were then compared to RNA-seq data and homologous sequences from other *Fusarium* species using BLAST 2.9.0+ software [44,45] and the exonerate (v2.2.0) program (http://www.ebi.ac.uk/~guy/exonerate/ accessed on 15 January 2021), respectively, to further validate the CDS data.

Functional annotation of the predicted amino acid sequences of the putative genes was then performed using EggNOG v5.0 software [46,47] to produce functional annotation associated with functional domains, Gene Ontology (GO) terms, and Kyoto Encyclopedia of Genes and Genomes (KEGG) pathways. A final analysis was then performed using BUSCO (v4.0.4) software [48,49,50] to verify the completeness of the genome assembly and protein annotation. In addition to the main analysis, non-coding genes were annotated using Rfam (14.4) software [51,52]. The mitochondrial genome was analyzed on the MITOS Webserver (http://mitos.bioinf.uni-leipzig.de/index.py accessed on 18 August 2021).

### 4.6. Annotation of Repetitive Sequences in the FG-12 Genome

Unique repetitive sequences in the FG-12 genome were identified using a self-blast search strategy performed using RepeatModeler (v 2.0.1) software (https://github.com/Dfam-consortium/RepeatModeler/releases accessed on 15 January 2021). Further analysis was then performed using RepeatMasker (v 4.1.0) software (http://www.repeatmasker.org/ accessed on 15 January 2021) to search for known repetitive sequences using a cross-match program comparing the unique repetitive sequences with a Repbase-derived RepeatMasker library.

### 4.7. Synteny Analysis Comparing the PH-1 and FG-12 Genomes

Homologous regions of the FG-12 and PH-1 genomes were identified using MUMmer (v3.1) software [53], while homologous co-ordinates were identified using NUCmer software [54]. The matching regions of the FG-12 scaffolds and the PH-1 chromosomes and mitochondrial DNA were then identified and visualized using gnuplot (v4.6.7) (https://sourceforge.net/projects/gnuplot/ accessed on 18 August 2021) and Genome Pari Rapid Dotter (gepard, v1.30) online (hppt://cube.univie.ac.at/gepard accessed on 18 August 2021), respectively.

### 4.8. Annotation of CAZyme Genes and Secondary Metabolite Clusters in the FG-12 Genome

Putative carbohydrate active enzyme (CAZyme) genes encoded by the FG-12 genome were identified using the CAZy annotation pipeline [55], while secondary metabolite (SM) clusters associated with the biosynthesis of fungal secondary metabolites were predicted using the antiSMASH 6.0 online tool [56]. Sequence alignment of the trichodiene-11-one gene cluster from FG-12 and PH-1 using the *F.* *asiaticum* cluster (BGC0001811) as a bridge sequence was performed using software [56].

### 4.9. Identification of Putative Effectors Encoded by the FG-12 Genome

Putative effectors were initially selected by searching the FG-12 CDS data for proteins that contained signal peptides but lacked transmembrane helices and GPI-anchors (ω-sites) using SignalP-5.0 [57], TMHMM Server v2.0 [58], and PredGPI software [59], respectively. Candidate sequences were then selected on the basis of protein size (≤500 amino acid residues) and number of cysteine residues (≥4) using the Perl programming language.

### 4.10. Functional Analysis of Candidate Effectors

The genes of the candidate effectors (with or without signal peptides) from *F. graminearum* FG-12 were amplified using the primer sets listed in Supplementary Table S10. The resulting PCR products were cloned into the pBinGFP2 expression vector using appropriate restriction enzymes and the ClonExpress II OneStep Cloning kit (Vazyme Biotech, Nanjing, China). The modified plasmids were then introduced into *Agrobacteria* strain GV3101 using the freeze–thaw method [60], and selection on Luria–Bertani (LB) medium containing 50 µg/mL kanamycin and rifampicin.

After verification, the *Agrobacteria* carrying the candidate effectors were inoculated in LB medium supplemented with kanamycin and rifampicin (50 µg/mL) and cultured overnight at 28 °C with shaking (150 rpm). The *Agrobacteria* cells were then collected and re-suspended in infiltration buffer (10 mM MES [pH 5.7], 10 mM MgCl2, and 150 µM acetosyringone) to a final OD_600_ of 0.6. After standing at room temperature for 2.5 h, the infiltration of *Agrobacteria* carrying different effector genes into the leaves of 3-week-old *N. benthamiana* plants using needleless syringes took place [61]. *Agrobacteria* carrying pBinGFP2 were used as the negative control (GFP), while the addition of Bax was used as a positive control (+Bax), with the symptoms resulting from infiltration being evaluated and photographed at 3 days post-inoculation (dpi).

DAB staining of the *N. benthamiana* leaves was also performed to detect hydrogen peroxide (H_2_O_2_) using 3,3′-diaminobenzidine (DAB)-HCl [62]. Briefly, excised leaves were immersed in a DAB solution (1 mg/mL, pH 3.8) at room temperature for 12 h in the presence of light, before being destained in 95% ethanol in a 100 °C water bath for 5 min.

## Figures and Tables

**Table 1:** Summary statistics for the *F. graminearum* FG-12 genome.

Criteria	FG-12
Genome size (bp)	35,949,582
Number of contigs	6
Contig N50 (bp)	7,835,986
Number of scaffolds	5
Scaffold N50 (bp)	8,894,400
Number of N50 scaffolds	2
Total coverage	220×
GC content	47.99%

**Table 2:** Summary statistics for the *F. graminearum* FG-12 nuclear genome annotation.

Criteria	FG-12
Predicted number of coding genes	12,470
Number of non-coding RNAs	354
Number of genes with exons	7256
Exon mean number per gene	2.4
Exon mean length	1398
Intron mean number per gene	1.4
Exon mean length	502
Number of gene amino acids < 100 aa	738
Number of truncated ORFs	1291

**Table 3:** Summary statistics for the *F. graminearum* FG-12 mitochondrial genome annotation.

Criteria	FG-12
Genome size (bp)	98,312
GC content	31.71%
Number of genes	59
Mean gene length (bp)	285
Number of PCGs	28
Number of tRNAs	27
Number of rRNAs	4

**Table 4:** Repetitive DNA profiles from the *F. graminearum* FG-12 genome and the PH-1 genome.

Type	FG-12			PH-1		
	Copy Number	Length (bp)	Percentage	Copy Number	Length (bp)	Percentage
Retroelements	303	343,994	0.96%	88	76,949	0.20%
SINEs:	40	4522	0.01%	50	5390	0.01%
LTR elements:	263	339,472	0.95%	38	71,559	0.19%
Gypsy/DIRS1	263	339,472	0.95%	38	71,559	0.19%
DNA transposons	-	-	0.00%	135	86,837	0.23%
Tc1-IS630-Pogo	-	-	0.00%	135	86,837	0.23%
Rolling circles	-	-	0.00%	16	2170	0.01%
Unclassified:	1574	184,287	0.51%	1547	194,631	0.51%
Total interspersed repeats:		528,281	1.47%		358,417	0.94%
Small RNA:	85	8303	0.02%	171	18,452	0.02%
Satellites:	66	7914	0.02%	0	0	0.02%
Simple repeats:	5690	229,406	0.64%	5824	231,111	0.64%
Low complexity:	611	28,533	0.08%	695	32,076	0.08%
Total		800,180	2.23%		639,598	1.68%

**Table 5:** Number of CAZymes encoded by the *F. graminearum* FG-12 and PH-1 genomes.

Type	FG-12	PH-1
AA	94	102
CBM	6	9
CE	41	44
GH	238	247
GT	100	98
PL	22	22
Total	501	522

## Data Availability

The entire genome of *F. graminearum* strain FG-12 has been deposited in the GenBank database under accession number PRJNA743144.

## Supplementary Materials

The supplementary materials referred to in the current study can be found at www.mdpi.com/2309-608X/7/9/699/s1, and include: Figure S1. Results of the k-mer analysis of the FG-12 genome using Jellyfish software; Figure S2. Results of the BUSCO analysis of the FG-12 genome; Figure S3. Go annotation of the FG-12 genome; Figure S4. KEGG annotation of the FG-12 genome; Figure S5. GO annotation of genes specific to the *F. graminearum* type strain PH-1; Figure S6. KEGG annotation of genes specific to the *F. graminearum* type strain PH-1; Figure S7. GO annotation of putative effectors in the FG-12 genome; Figure S8. KEGG annotation of putative effectors in the FG-12 genome; Figure S9. Transient expression of putative effectors in *N. benthamiana* leaves via agro-infiltration; Figure S10. Sequence alignment of the pathogenicity-related ribosomal nuclease gene from *F. graminearum* FG-12 and PH-1; Figure S11. Dot plot diagram comparing the structure of the mitochondrial (Mit) genomes of *F. graminearum* FG-12 and PH-1; Figure S12: Phylogenetic tree constructed using MEGA-X based on the genomes of 11 filamentous fungal strains; Table S1. *F. graminearum* genomes listed in NCBI database; Table S2. Genome survey data produced by k-mer analysis of the FG-12 genome using Jellyfish software; Table S3. Summary statistics from protein annotation of the FG-12 genome; Table S4. Summary statistics from non-coding RNA annotation of the FG-12 genome; Table S5. Repetitive DNA profiles from the *F. graminearum* FG-12 genome and *M. oryzae* genome; Table S6. Genes specific to the *F. graminearum* type strain PH-1; Table S7. Annotation of genes specific to the *F. graminearum* type strain PH-1; Table S8. Putative effectors encoded by the FG-12 genome; Table S9. Annotations of putative effectors encoded by the FG-12 genome; Table S10. Primers used to amplify full-length gene sequences of candidate effectors encoded by the FG-12 genome; Table S11. *Fusarium* species referenced during the genome annotation; Table S12. Information of the filamentous fungal strains used in genome phylogenetic analysis; Table S13. Number of putative effector proteins encoded by the *F. graminearum* FG-12 and PH-1 genomes.
