# Supplementary material for: Genome Sequence Analysis of the Fungal Pathogen Fusarium graminearum Using Oxford Nanopore Technology
Source: J Fungi (Basel). 2021 Aug 27;7(9):699. doi: 10.3390/jof7090699 (PMC8465144; doi:10.3390/jof7090699)
Supplement: Supplementary file 1 [file jof-07-00699-s001.zip › jof-1347198-supplementary Figures.pdf]

Journal of
Fungi

MDPI

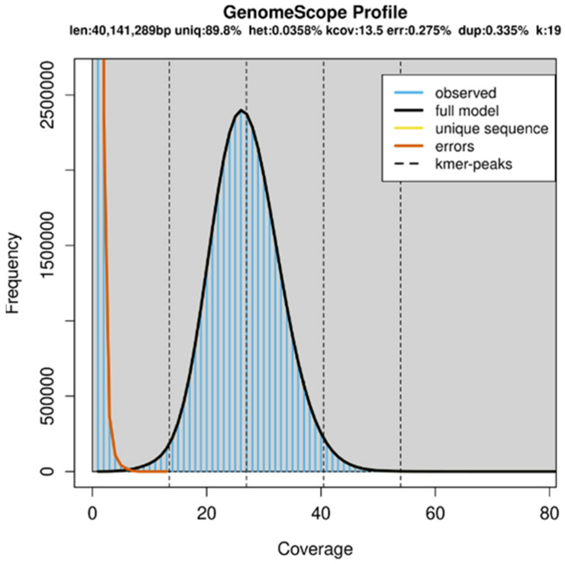

Figure S1. Results of the k-mer analysis of the FG-12 genome using Jellyfish software.

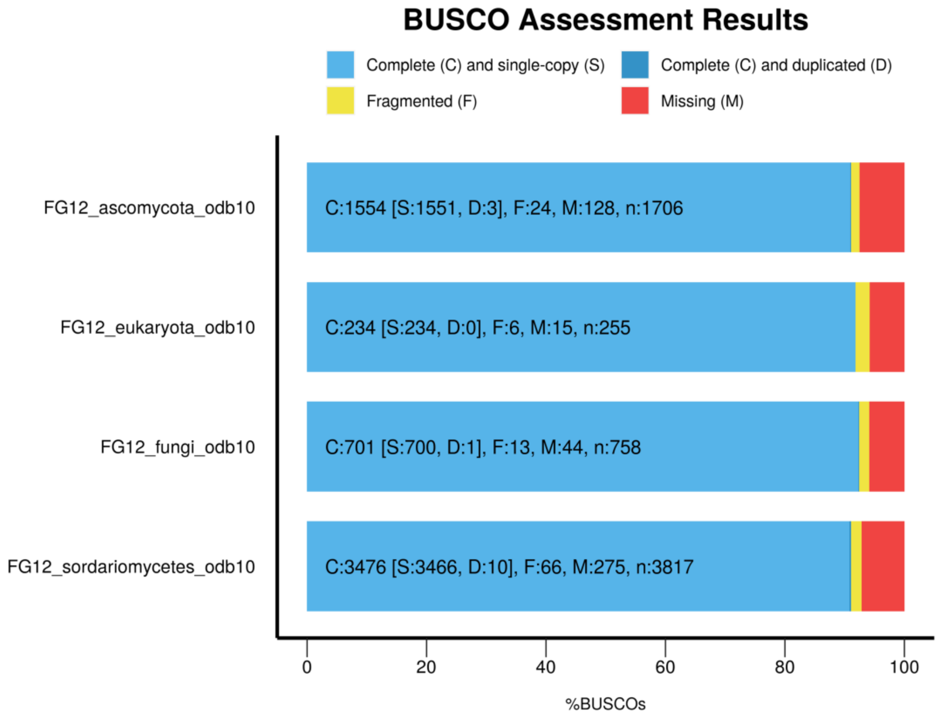

Figure S2. Results of the BSUCO analysis of the FG-12 genome.

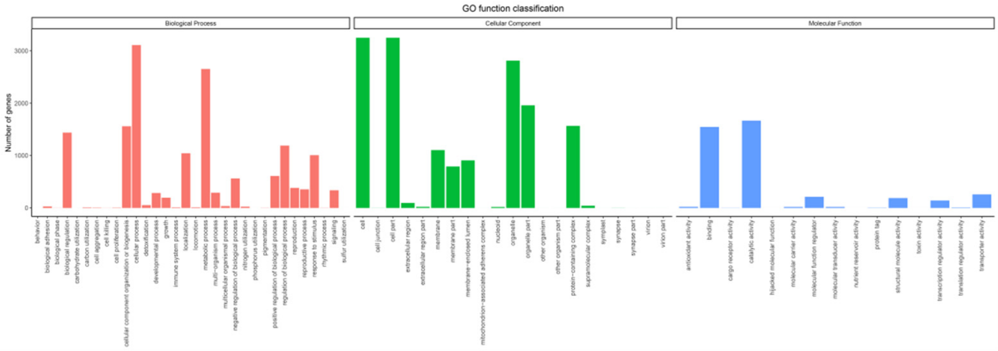

Figure S3. Go annotation of the FG-12 genome.

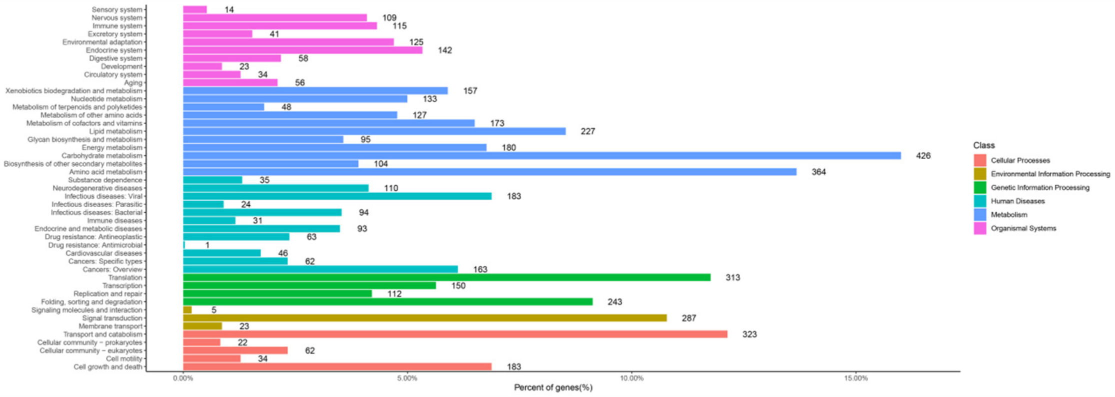

Figure S4. KEGG annotation of the FG-12 genome.

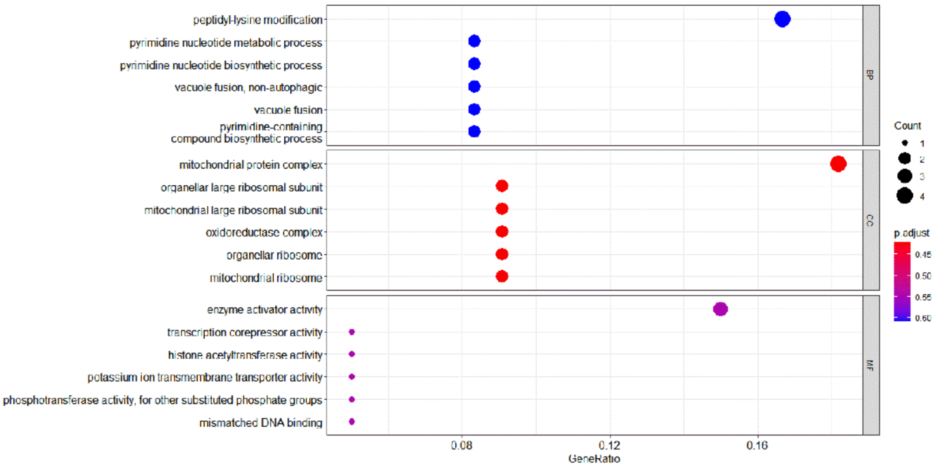

Figure S5. GO annotation of genes specific to the *F. graminearum* type strain PH-1.

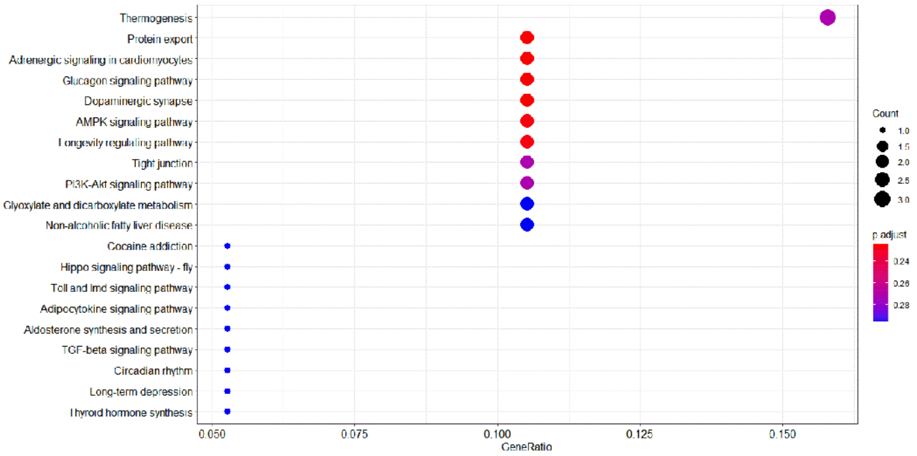

Figure S6. KEGG annotation of genes specific to the *F. graminearum* type strain PH-1.

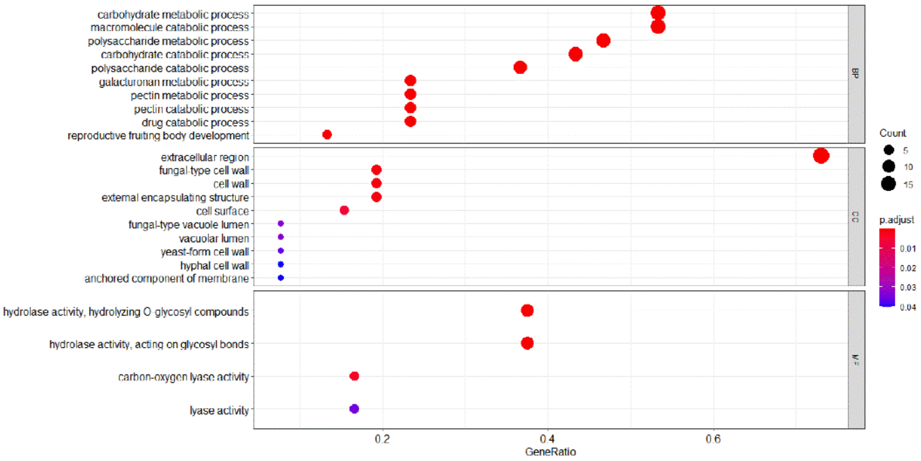

Figure S7. GO annotation of putative effectors in the FG-12 genome.

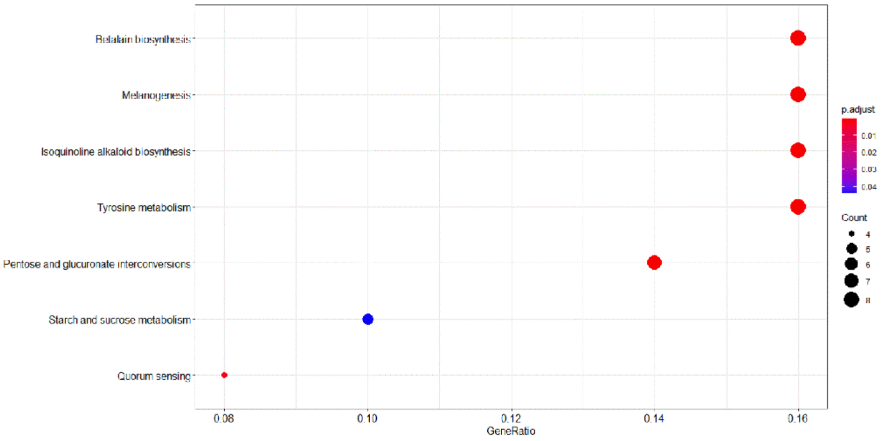

Figure S8. KEGG annotation of putative effectors in the FG-12 genome.

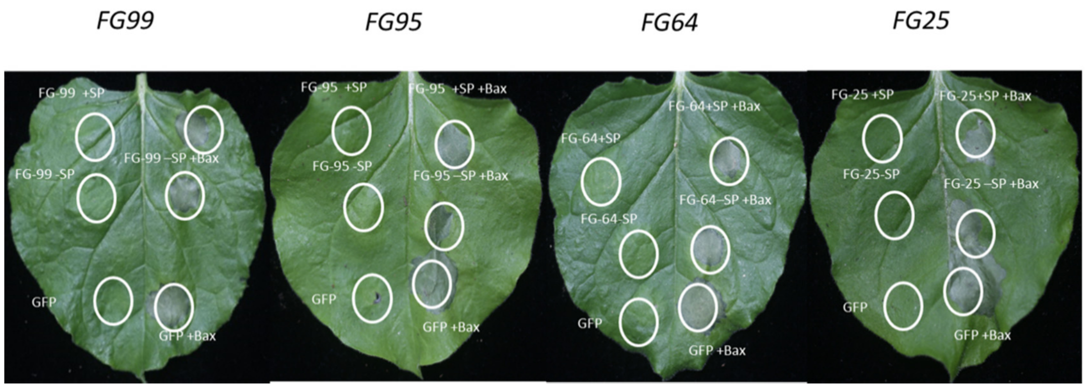

Figure S9. Transient expression of putative effectors in *N. benthamiana* leaves via agro-infiltration. Symptoms on leaves of *N. benthamiana* were assessed at 3 days post–inoculation (dpi). Putative effectors including FG99 (FGMG_005117), FG95 (FGMG_005015), FG64 (FGMG_001857), FG25 (FGMG_006174), while *Agrobacteria* carrying the unaltered pBinGFP2 vector was used as a negative control (GFP), and the addition of Bax as a positive control (+Bax).

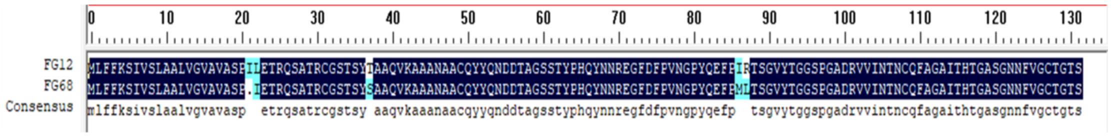

Figure S10. Sequence alignment of the pathogenicity-related ribosomal nuclease gene from *F. graminearum* FG-12 and PH-1. FG12 indicates the predicted amino acid sequence from the CDS of the type strain PH-1, while FG68 indicates the equivalent CDS from FG-12.

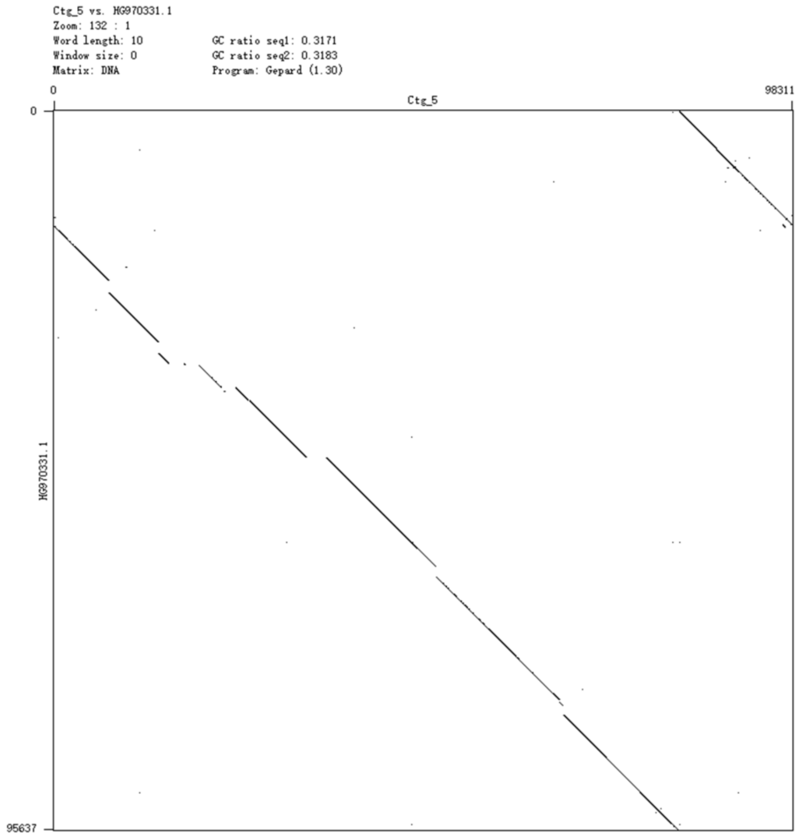

Figure S11. Dot plot diagram comparing the structure of the mitochondrial (Mit) genomes of *F. graminearum* FG-12 and PH-1. Complete (Mit) genome alignment using Gepard software was used to compare the FG-12 Mit genome (Ctg_5) listed on the x-axis to PH-1 Mit genome (HG970331.1) on the y-axis. If the two sequences were perfectly identical, a single lines would dissect the graph from the top left to the bottom right.

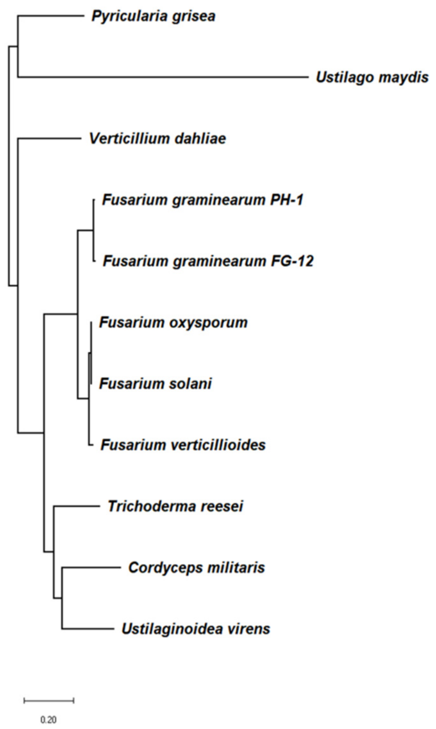

Figure S12. Phylogenetic tree constructed using MEGA-X (https://www.megasoftware.net/) based on the genomes of 11 filamentous fungal strains. The tested strains included *Fusarium graminearum* FG12 and PH-1, *Fusarium verticillioides, Fusarium solani, Fusarium oxysporum, Ustilago maydis, Pyricularia grisea, Verticillium dahliae, Ustilaginoidea virens, Trichoderma reesei,* and *Cordyceps militaris*.
